# Supplementary material for: A Global Screen for Assembly State Changes of the Mitotic Proteome by SEC-SWATH-MS
Source: Cell Syst. 2020 Feb 26;10(2):133–155.e6. doi: 10.1016/j.cels.2020.01.001 (PMC7042714; doi:10.1016/j.cels.2020.01.001)

Q9BSJ8 | ESYT1_HUMAN | ESYT1 FAM62A KIAA0747 MBC2
Monomer MW [kDa]: 122.856 Monomer expected elution fraction: 38

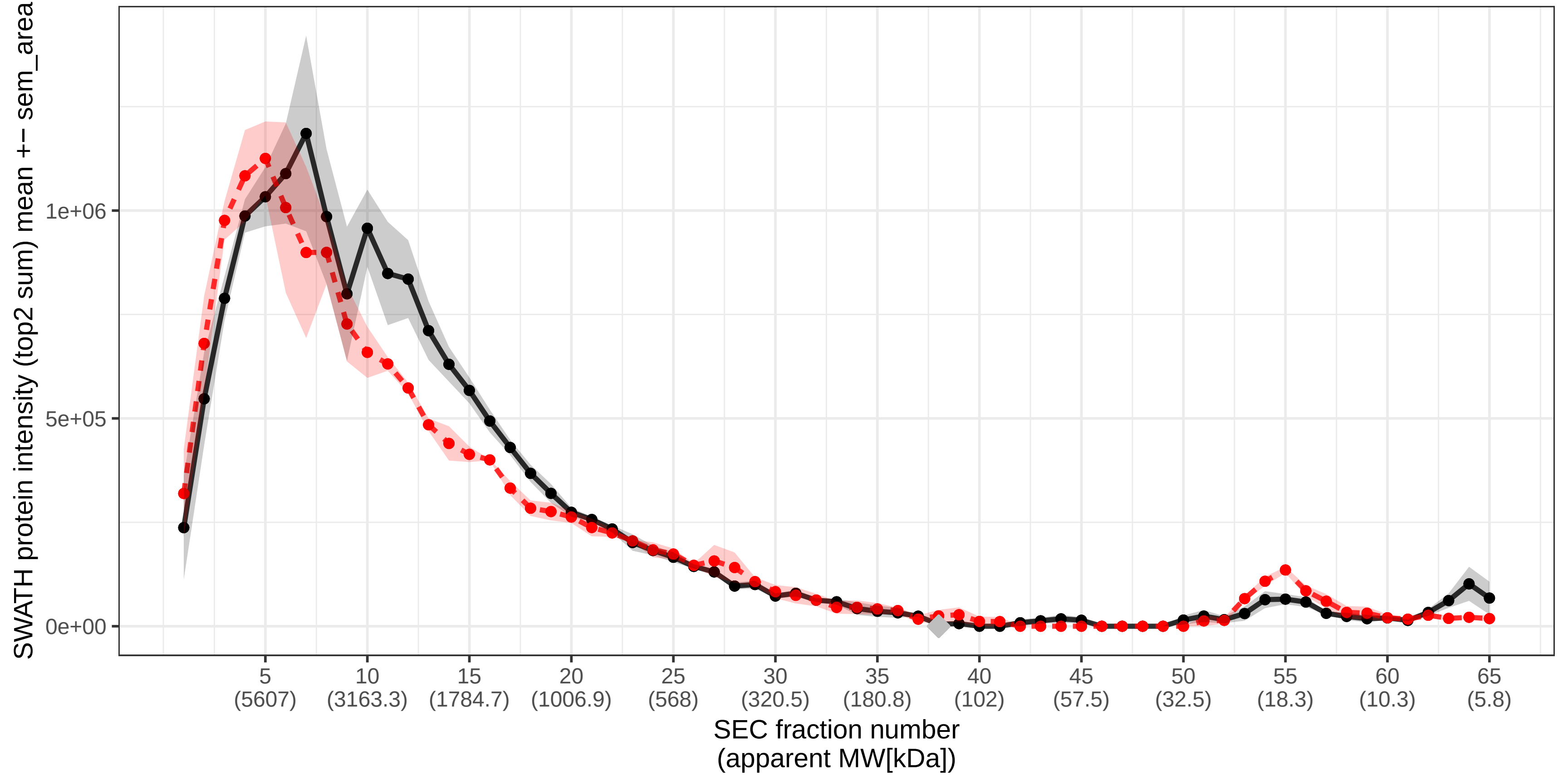

Supplement: Data S1. SEC-SWATH-MS Protein Chromatograms, Related to Figure 1 [file mmc6.zip › SECchrom_Q9BSJ8_ESYT1_HUMAN_ESYT1_FAM62A_KIAA0747_MBC2.pdf]
